# Evaluation of standardized DICOM labels assigned by a hybrid AI tool and its impact on radiologists’ reading times

**DOI:** 10.1007/s00330-026-12670-w

**Published:** 2026-06-10

**Authors:** Bina Tariq, Meike W. Vernooij, Ken Redekop, Daniel Bos, Jacob J. Visser

**Affiliations:** 1 Department of Radiology & Nuclear Medicine, Erasmus MC, Rotterdam, The Netherlands; 2 Department of Epidemiology, Erasmus University Medical Centre, Rotterdam, The Netherlands; 3 Erasmus School of Health Policy and Management, Erasmus University Rotterdam, Rotterdam, The Netherlands

**Keywords:** Artificial intelligence, DICOM metadata, Reading time, Standardization, Workflow

## Abstract

**Objectives:**

Medical artificial intelligence (AI) tools are increasingly used to manage the growing imaging workload in radiology. Although highly relevant to daily clinical practice, AI-driven workflow tools remain underrepresented in research. Inconsistencies in DICOM metadata are a major obstacle to workflow optimization, AI integration, and inter-institutional data sharing. Automatic DICOM metadata standardization is an important step towards addressing these challenges. This study evaluates the labeling accuracy of a commercially available AI-aided hybrid software tool and its impact on radiologists’ reading times.

**Materials and methods:**

A retrospective cohort study assessed the labeling accuracy of the DICOM standardization tool. A retrospective before-and-after design evaluated its impact on reading times. The tool was applied by a radiology provider between 2022 and 2024. Standardized DICOM labels (modality, body part, laterality, plane, contrast protocol) across 422 CR images and 1503 CT series were manually reviewed (gold standard). In a separate analysis, reading times before (10,966 cases) and after (10,342 cases) DICOM standardization were compared.

**Results:**

Labeling accuracy ranged from 83 to 100% for body part, 91 to 100% for plane, and 88–100% for protocol classification. Following implementation, average reading times significantly decreased for CT Abdomen (−2.9 min), Total Body (−2.2 min), Head (−0.73 min), and Temporal Bone (−2.5 min) (*p* ≤ 0.02), with relative efficiency gains of 8–22%. Extrapolated annually, this equals 270 h saved. No significant changes were observed for CT Chest and Sinus/Orbits.

**Conclusion:**

This study suggests that an AI-aided tool can adequately standardize DICOM labels and may be associated with statistically significant reductions in radiologists’ reading times following implementation.

**Key Points:**

***Question*** *Inconsistent DICOM metadata pose long-standing challenges in imaging data management, hindering processes such as workflow efficiency, AI integration, and inter-institutional image sharing. What is the role of AI solutions to address DICOM inconsistencies*?

***Findings*** *An AI-aided hybrid software tool adequately standardized DICOM labels and may be associated with statistically significant reductions in radiologists’ reading times following implementation*.

***Clinical relevance*** *Standardizing DICOM metadata may help streamline radiology workflows and image accessibility, benefiting both radiologists and other healthcare professionals*.

**Graphical Abstract:**

**Figure:**
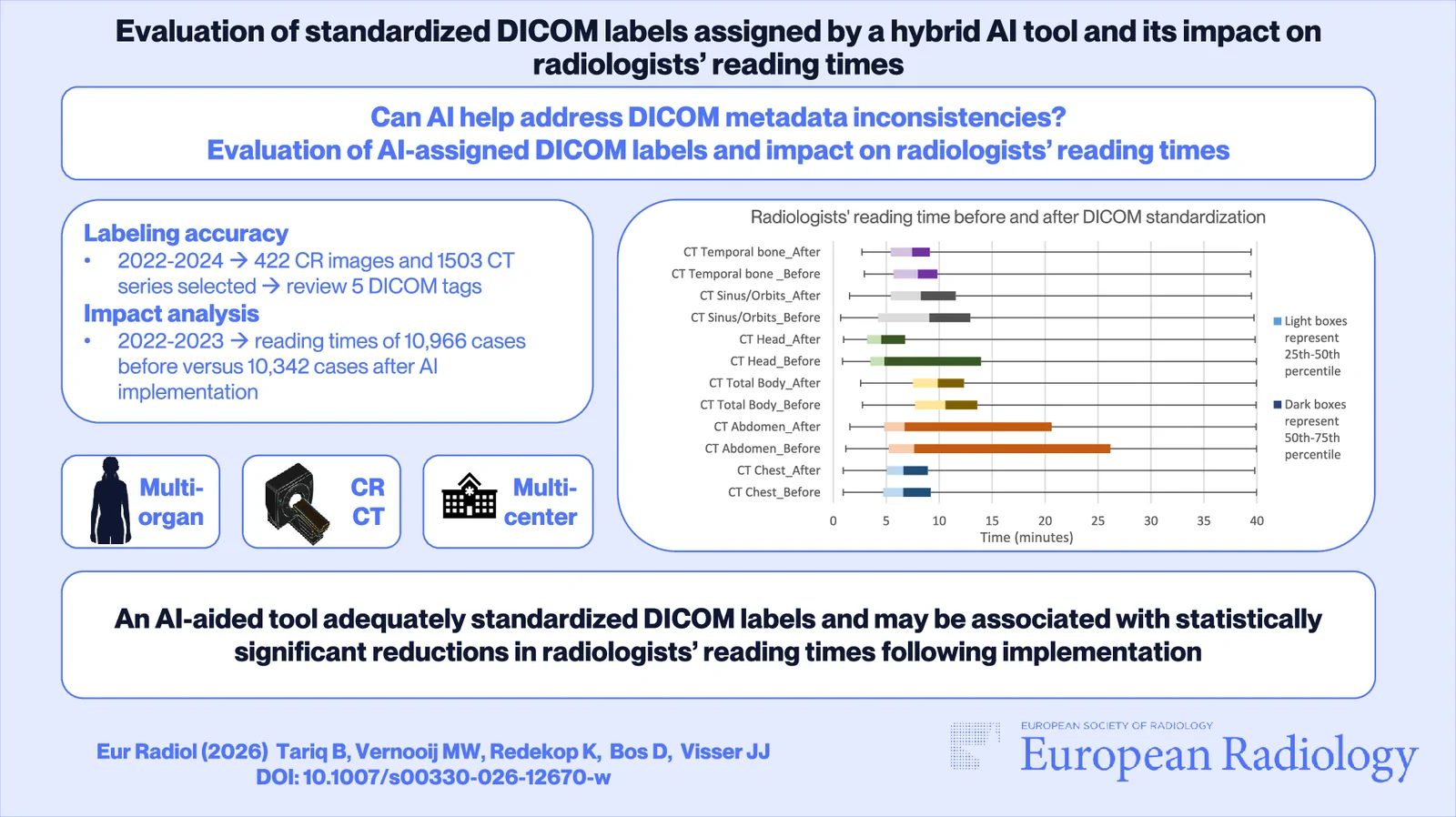

## Introduction

The field of radiology is experiencing a continuous increase in the volume of imaging studies, necessitating efficient workflows to manage this growth. In response to the corresponding rise in workload, artificial intelligence (AI) tools are being increasingly implemented to assist radiologists [1, 2]. However, the focus has primarily been on triage and pathology detection [3–9]. AI-driven workflow improvement tools have received comparatively little attention [4, 7, 8, 10–13], despite radiology’s heavy reliance on (workflow) standards [10], such as the universal Digital Imaging and Communications in Medicine (DICOM). DICOM serves as the driving force behind image acquisition, storage, interpretation, and transfer across various systems and devices [7, 11, 14–20].

Managing imaging data through DICOM has historically posed challenges. Previous studies have highlighted metadata inconsistencies as a recurring issue, often resulting from vendor-specific differences and inconsistent application of DICOM standards across modalities, protocols, and institutions [11, 14, 17, 21]. These inconsistencies complicate the integration and processing of imaging data into picture archiving and communication systems (PACS) and radiology information systems (RIS)/hospital information systems (HIS), thereby contributing to workflow inefficiencies [18–20, 22]. Moreover, inconsistent image labeling hinders prior image retrieval to generate Standard or Smart Display Protocols (SDPs), which impairs comparative analysis or data acquisition for research purposes [5, 7, 11, 18]. As the integration of all AI applications into PACS ecosystems also depends on consistent metadata [1, 5, 7, 10, 14, 22], variability across institutions further exacerbates these challenges, especially with the increasing need for inter-institutional data sharing [19, 22, 23].

Emerging AI tools may offer an opportunity to target these foundational DICOM metadata issues before their integration into PACS [3, 5, 7, 8, 12, 13, 23], potentially leading to an improved ecosystem [7, 8, 11] and increased workflow efficiency, e.g., through enhanced hanging protocols/SDPs, improved integration of AI tools, interoperability across diverse systems [3, 10, 13]. Nevertheless, no studies could be found on AI-based solutions to improve DICOM metadata, highlighting a critical gap in the field [5, 7, 11]. Therefore, this study aims to evaluate a commercially available hybrid AI tool that generates standardized DICOM labels and to assess its impact on radiologists’ reading times (RTs).

## Methods

### Study design

This study was approved by the institutional review board (IRB) of the Erasmus Medical Centre (METC-number: MEC-2023-0534), and informed consent was waived due to the observational and retrospective nature of this study.

A teleradiology provider serving multiple clinical institutions, including approximately 40 National Health System (NHS) trusts in the United Kingdom (UK), implemented an AI-aided tool across its PACS environment. This tool standardized the DICOM metadata of radiology studies received from various institutions. A pooled dataset was created by consecutively selecting studies without stratification by hospital. All data were anonymized before being sent to researchers at an academic hospital in the Netherlands for independent analysis.

This study consists of two parts, each with a distinct design. The first part is a retrospective cohort study exploring the labeling accuracy of an AI tool that standardizes DICOM metadata. The second part is a retrospective before-and-after design assessing the impact of AI-aided DICOM standardization on radiologists’ RTs. Both parts are described below.

#### Labeling accuracy

Eight imaging study types were chosen to be analyzed: computed radiography (CR) chest, CR cervical spine (C-Spine), CR knee, computed tomography (CT) chest, CT abdomen, CT total body, CT head, and CT face (see Supplementary Table 1).

These groups were selected to reflect commonly encountered study types in routine radiology practice, covering a broad range of anatomical regions. Five DICOM labels were selected from a larger pool for their relevance to daily clinical workflow based on the expert opinion of the authors. These DICOM labels were reviewed for each imaging group if applicable: modality, body part, plane, laterality, and protocol (contrast enhancement status).

Power calculations were proposed by an experienced statistician to estimate a meaningful sample size for exploring labeling accuracy. These calculations were based on the inclusion of 60 datapoints per DICOM metadata layers, resulting in 120 datapoints for CR Knee and CR Chest each, 180 for CR C-Spine, and 300 for each CT. Accordingly, a total of 422 CR images (227 cases) and 1503 CT series (421 cases) were consecutively selected from an anonymized and randomized pool between 2022 and 2024.

The AI-assigned DICOM labels were manually reviewed by a senior radiology resident (gold standard). In case of uncertainty, a senior radiologist with 15 years of clinical experience was consulted. Labels were verified against the visual content of the studies, rather than assigned blindly by the reviewer. This approach was chosen due to the exploratory nature of the analysis and to reflect real-world clinical practice.

#### Impact analysis

We furthermore assessed the impact of AI-aided DICOM standardization on RTs. RT was defined as the time from opening a case until closing it with a finalized report. These RT data were automatically registered in PACS as part of the normal workflow of the teleradiology provider. All consecutive CT studies performed between January 2022 and August 2023 with RTs ranging from 0 (including studies < 60 s) to 40 min were selected for inclusion. Studies with RTs exceeding 40 min were identified as outliers and excluded. Additional exclusion criteria were studies without a recorded date, flagged/ prioritized studies, data from specific clients (e.g., lung cancer screening), and data from the Christmas holiday period due to connectivity and server issues. CR studies were excluded from this analysis due to their inherently short RTs and coverage with other AI tools.

A total of 28,067 CT scans were identified, of which 24,269 cases met the inclusion criteria and were chronologically categorized into three periods:

- Before AI-aided DICOM standardization (January–September 2022): 10,966 cases.
- Transition period (October–November 2022): 2961 cases.
- After AI-aided DICOM standardization (December 2022–August 2023): 10,342 cases.

**Fig. 1:**
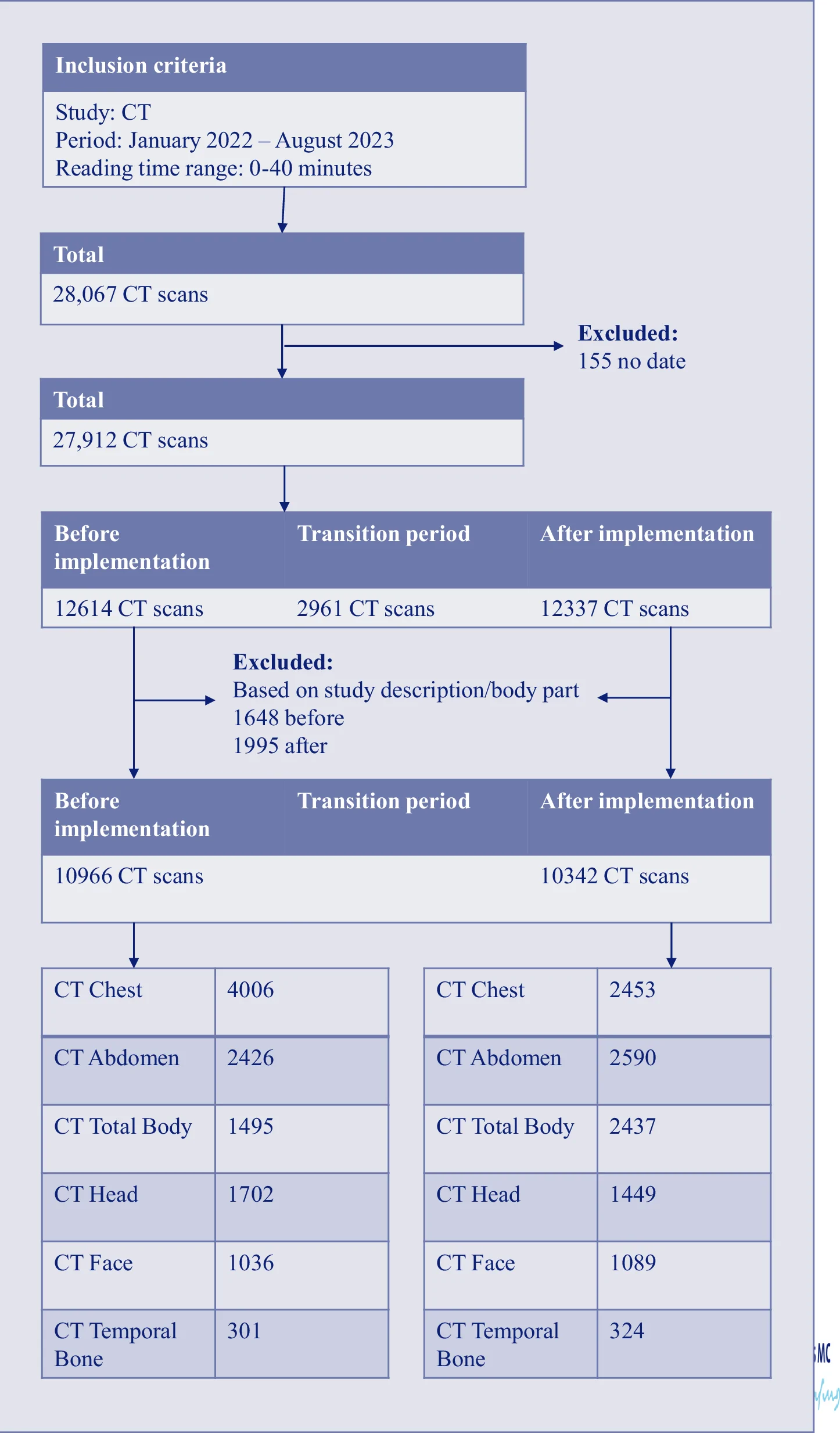
Flowchart of the case selection for the impact analysis on RTs before and after AI-aided DICOM standardization

The AI tool was introduced in October 2022, with two months designated as a transition period to allow radiologists to adapt. Cases from December 2022 onward were classified as part of the “after” period. Six groups were identified from the dataset (see Supplementary Table 2), representing commonly encountered study types in routine radiology practice, covering a broad range of anatomical regions: CT chest (*N* = 4006 before, *N* = 2453 after), CT abdomen (*N* = 2426 before, *N* = 2590 after), CT total body (*N* = 1495 before, *N* = 2437 after), CT head (*N* = 1702 before, *N* = 1449 after), CT face (*N* = 1036 before, *N* = 1089 after), and CT temporal bone (*N* = 301 before, *N* = 324 after) (see Fig. 1).

### Software

Within the existing workflow of a teleradiology provider, a commercially available software tool was installed that standardizes all DICOM tags prior to PACS ingestion by mapping inconsistent, incomplete, or non-standard entries to a consistent ontology. The tool combines rule-based preprocessing of DICOM tags with deep learning-based analysis of metadata and pixel data to generate standardized DICOM labels. This includes parsing existing metadata (e.g., modality, study description, series description, body part examined), analyzing pixel data using deep learning to identify, for example, anatomical regions, scan planes, and contrast phases when metadata is incomplete, and aligning results with controlled vocabularies for semantic consistency. The proportion of rule-based vs AI-based processing can vary for each DICOM tag, depending on metadata inconsistency [24–26]. A detailed description of the algorithmic framework is provided in the Supplementary Material.

The labeling accuracy was evaluated when module 2.1 of the software was in use. The data for the impact analysis was extracted before a software update when module 1.3 was in use. Access to the anonymized data was provided to the researchers through a secure virtual PACS environment.

### Statistical analysis

All statistical analyses were designed and reviewed in consultation with an experienced statistician.

#### Labeling accuracy

The evaluation of AI-aided DICOM labels was conducted at both the case level and the image level. A case referred to a single radiology study performed on a patient with a unique accession number and including all acquired views (for CR studies) or series and reconstructions (for CT). An image referred to an individual radiographic projection (for CR studies) or a single series/reconstruction (for CT studies) within a case. For each DICOM label (modality, body part, plane, and protocol), the number of correctly labeled cases and images was evaluated. Labeling accuracy was expressed as the percentage of correctly labeled cases and images out of the total number of cases and images within the corresponding group, respectively. Labeling accuracy analysis was exploratory in nature, utilizing purely descriptive statistics without applying sensitivity or specificity calculations. The intent was not to estimate accuracy with specific precision or conduct hypothesis testing, and no predefined accuracy thresholds or margin of error calculations were applied.

#### Impact analysis

The impact of AI-aided DICOM standardization on RT was assessed using descriptive statistics and the Mann–Whitney *U*-test, a non-parametric test suited for comparing two independent groups with non-normally distributed data. For each CT study group, the number of cases, average RT, median RT, standard deviation, and interquartile ranges (25th and 75th percentiles) were calculated before and after AI-aided DICOM standardization.

The efficiency gain for each CT category was calculated both as the absolute difference in average RT before and after implementation, and as the relative percentage reduction in average RT, using the following formula:

$$\frac{{average}\,{reading}\,{time}\,{before}\,\left(\min \! .\right)-{average}\,{reading}\,{time}\,{after}\,(\min \! .)}{{average}\,{reading}\,{time}\,{before}\,\left(\min \! .\right)}\,x\,100 \% .$$

The annual case volume for each CT category was calculated by dividing the total number of cases before and after implementation over an 18-month period by 12 months. The absolute efficiency gain was then multiplied by the annual case volume to estimate the potential total annual efficiency gain for each CT category. The total estimated annual time savings was derived by summing the efficiency gains across all CT categories. The Mann–Whitney *U*-test was applied to evaluate statistical differences in RTs between the two phases, with a significance level set at *p*-value < 0.05.

## Results

### Labeling accuracy

#### CR studies

For chest radiographs, all reviewed DICOM labels were 100% accurate (113/113 cases and 120/120 images) (see Table 1). For C-spine radiographs, modality and body part labels were 100% accurate (48/48 cases and 120/120 images). Plane labels were 94% accurate at the case level (45/48) and 98% at the image level (117/120), with high accuracy for frontal (98%) (46/47) and odontoid (100%) views (12/12). Lateral projections showed minor variation of 97% (59/61), with three mislabeled as mandible oblique (*n* = 2) and frontal (*n* = 1) views (see Table 1). For knee radiographs, modality and body part labels were 100% accurate (66/66 cases and 182/182 images). Plane labels were 91% accurate at the case level (60/66) and 96% at the image level (175/182). Seven lateral knee images were mislabeled as skyline (4), oblique (2), and frontal (1). Among these, one contained a knee prosthesis, and five had a horizontal beam orientation. Laterality labels were 100% accurate for both right-sided (31/31 cases and 90/90 images) and left-sided studies (35/35 cases and 92/92 images). Notably, three cases contained posteroanterior frontal views of the left knee, which may be misinterpreted as a right knee by an inexperienced observer or in a hasty evaluation. However, the AI tool correctly identified and labeled these as left knees.

**Table 1:** Labeling accuracy of an AI-aided DICOM standardization tool for CR studies

		Chest		C-Spine^a^		Knee	
		Cases^b^ (*N*/*n*) [%]^c^	Images^d^ (*N*/*n*) [%]	Cases (*N*/*n*) [%]	Images (*N*/*n*) [%]	Cases (*N*/*n*) [%]	Images (*N*/*n*) [%]
Modality	CR	113/113 [100.0]	120/120 [100.0]	48/48 [100.0]	120/120 [100.0]	66/66 [100.0]	182/182 [100.0]
Body Part		113/113 [100.0]	120/120 [100.0]	48/48 [100.0]	120/120 [100.0]	66/66 [100.0]	182/182 [100.0]
Plane	Total	113/113 [100.0]	120/120 [100.0]	45/48 [93.8]	117/120 [97.5]	60/66 [90.9]	175/182 [96.2]
	Frontal	-	120/120 [100.0]	-	46/47 [97.9]	-	69/70 [98.6]
	Lateral	-	-	-	59/61 [96.7]	-	64/66 [97.0]
	Odontoid	-	-	-	12/12 [100.0]	-	-
	Skyline/axial	-	-	-	-	-	42/46 [91.3]
Laterality	Total	-	-	-	-	66/66 [100.0]	182/182 [100.0]
	Right	-	-	-	-	31/31 [100.0]	90/90 [100.0]
	Left	-	-	-	-	35/35 [100.0]	92/92 [100.0]

^a^ C-spine: cervical spine

^b^ Case: a complete radiographic study with a unique accession number and including all acquired views

^c^ *N*/*n*: number of correctly labeled cases or images/total number of cases or images; percentages indicate labeling accuracy

^d^ Image: a single radiographic projection within a case

Overall, AI-aided DICOM labeling of CR studies was highly accurate, with 100% accuracy for modality and body part labeling and with minor variations in specific projections, particularly in C-spine lateral and knee skyline/axial views.

#### CT chest

The modality labels were 100% accurate across all cases (105/105) and images (299/299) (Table 2). The body part label was correctly identified in 96% of cases (94/98) and 95% of images (230/243), with a small proportion of the total number of cases remaining unlabeled (7/105 cases (7%), 56/299 (19%)). The plane label demonstrated 100% accuracy across all images (299/299), with axial (237/237), coronal (39/39), and sagittal (23/23) series correctly categorized. The distinction between non-contrast (59/59 cases, 128/128 images) and contrast-enhanced images (46/46 cases, 171/171 images) was 100% accurate. However, the specification of the contrast enhancement phase showed slightly lower accuracy (33/46 cases (72%), 142/171 (83%)). Therefore, the overall accuracy of protocol labeling was 88% at the case level (92/105) and 90% at the image level (270/299).

**Table 2:** Labeling accuracy of an AI-aided DICOM standardization tool for CT chest, abdomen, and total body

		Chest		Abdomen		Total Body	
		Cases^a^ (*N*/*n*) [%]^b^	Images^c^ (*N*/*n*) [%]	Cases (*N*/*n*) [%]	Images (*N*/*n*) [%]	Cases (*N*) [%]	Images (*N*) [%]
Modality	CT	105/105 [100.0]	299/299 [100.0]	159/159 [100.0]	300/300 [100.0]	42/42 [100.0]	304/304 [100.0]
Body part	Total	94/98 [95.9]	230/243 [94.7]	132/132 [100.0]	269/269 [100.0]	33/40 [82.5]	256/290 [88.3]
	Unlabeled	7/105 [6.7]	56/299 [18.7]	27/159 [17.0]	31/300 [10.3]	2/42 [4.8]	14/304 [4.6]
Plane	Total	105/105 [100.0]	299/299 [100.0]	159/159 [100.0]	300/300 [100.0]	42/42 [100.0]	304/304 [100.0]
	Axial	-	237/237 [100.0]	-	252/252 [100.0]	-	183/183 [100.0]
	Coronal	-	39/39 [100.0]	-	38/38 [100.0]	-	76/76 [100.0]
	Sagittal	-	23/23 [100.0]	-	10/10 [100.0]	-	45/45 [100.0]
Protocol	Total	92/105 [87.6]	270/299 [90.3]	158/159 [99.4]	299/300 [99.7]	42/42 [100.0]	304/304 [100.0]
	NON-CON^d^	59/59 [100.0]	128/128 [100.0]	12/12 [100.0]	19/19 [100.0]	2/2 [100.0]	10/10 [100.0]
	CE^e^	46/46 [100.0]	171/171 [100.0]	147/147 [100.0]	281/281 [100.0]	40/40 [100.0]	294/294 [100.0]
	CE phase^f^	33/46 [71.7]	142/171 [83.0]	146/147 [99.3]	280/281 [99.6]	40/40 [100.0]	294/294 [100.0]

^a^ Case: a complete CT study with a unique accession number and including all acquired series and reconstructions

^b^ *N*/*n*: number of correctly labeled cases or images/total number of cases or images; percentages indicate labeling accuracy

^c^ Image: a single series or reconstruction within a case

^d^ NON-CON: non-contrast enhanced

^e^ CE: contrast enhanced; presence of intravenous contrast

^f^ CE phase: specification of phase of contrast enhancement within the CE group; arterial phase, (portal)venous phase. Accuracy as a percentage of the CE group

#### CT abdomen

For CT Abdomen studies, modality labeling was 100% accurate (159/159 cases, 300/300 images) (see Table 2). The body part label achieved 100% accuracy at both the case (132/132) and image level (269/269). In 17% of cases (27/159) and 10% of images (31/300), a body part label was missing. The plane label showed 100% accuracy across axial (252/252), coronal (38/38), and sagittal (10/10) images. The distinction between non-contrast and contrast-enhanced images was 100% accurate (19/19 and 281/281, respectively), and the specification of contrast enhancement phase was also very highly accurate at 99.6% (280/281 images). So, protocol labeling showed high overall accuracy of 99% at the case level (158/159) and 99.7% at the image level (299/300).

#### CT total body

For CT Total Body studies, modality labeling was 100% accurate (42/42 cases, 304/304 images) (see Table 2). Body part labels were correctly assigned in 83% of cases (33/40) and 88% of images (256/290), with some studies remaining unlabeled (2/42 (5%) cases, 14/304 (5%) images). The plane and protocol labels were 100% accurate across all cases (42/42) and images (304/304).

#### CT head

For CT Head studies, modality labeling was 100% accurate across all cases (42/42) and images (297/297) (see Table 3). The body part label showed an accuracy of 88% at the case level (36/41) and 93% at the image level (270/290). One case had no body part label (7/297 (2%)). The plane labels were 100% accurate across all axials (152/152), coronal (81/81), and sagittal (68/68) images. Protocol labeling accuracy was 98% (41/42 cases, 290/297 images), with one non-contrast study (7 images) not correctly labeled.

**Table 3:** Labeling accuracy of an AI-aided DICOM standardization tool for CT head and facial bones

		Head		Facial bones	
		Cases^a^ (*N*) [%]^b^	Images^c^ (*N*) [%]	Cases (*N*) [%]	Images (*N*) [%]
Modality	CT	42/42 [100.0]	297/297 [100.0]	73/73 [100.0]	303/303 [100.0]
Body part	Total	36/41 [87.8]	270/290 [93.1]	67/67 [100.0]	281/281 [100.0]
	Unlabeled	1/42 [2.4]	7/297 [2.4]	6/73 [8.2]	22/303 [7.3]
Plane	Total	42 [100.0]	297/297 [100.0]	73/73 [100.0]	303/303 [100.0]
	Axial	-	152/152 [100.0]	-	197/197 [100.0]
	Coronal	-	81/81 [100.0]	-	76/76 [100.0]
	Sagittal	-	68/68 [100.0]	-	30/30 [100.0]
Protocol	Total	41/42 [97.6]	290/297 [97.6]	73/73 [100.0]	303/303 [100.0]
	NON-CON^d^	40/41 [97.6]	258/265 [97.4]	72/72 [100.0]	299/299 [100.0]
	CE^e^	5/5 [100.0]^f^	32/32 [100.0]	1/1 [100.0]	4/4 [100.0]

^a^ Case: a complete CT study with a unique accession number, and including all acquired series and reconstructions

^b^ *N*/*n*: number of correctly labeled cases or images/total number of cases or images; percentages indicate labeling accuracy

^c^ Image: a single series or reconstruction within a case

^d^ NON-CON: non-contrast enhanced

^e^ CE: contrast enhanced; presence of intravenous contrast

^f^ Four CT head studies included both non-contrast and contrast-enhanced images, while one case contained only a contrast-enhanced series

#### CT facial bones

For CT facial bones studies, 100% accuracy was achieved in modality labeling across all cases (73/73) and images (303/303). The body part label was 100% correctly assigned in all cases (67/67) and images (281/281), excluding six cases (8%) with missing body part labels. The plane labels were 100% accurate in axial (197/197), coronal (76/76), and sagittal (30/30) views. Protocols were also 100% correctly labeled (73/73 cases, 303/303 images).

Overall, AI-aided DICOM standardization for CT studies demonstrated high labeling accuracy, with minor variability in body part labels and contrast enhancement phase labels.

### Impact analysis

**Fig. 2:**
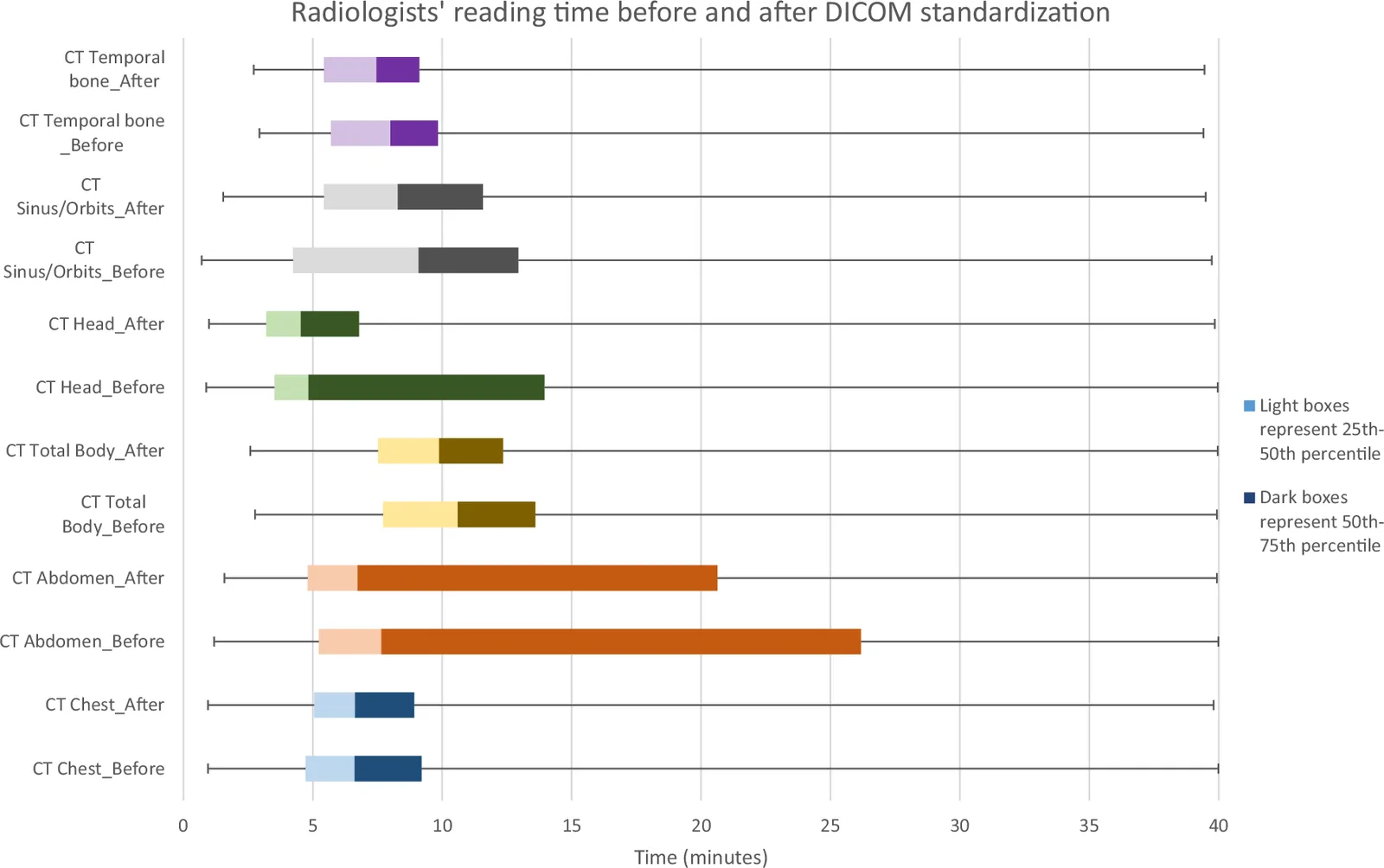
RTs of radiology studies before and after AI-aided standardization of the DICOM metadata

Implementation of the AI-aided DICOM standardization tool resulted in statistically significant reductions in RTs for CT Abdomen, CT Total Body, CT Head, and CT Temporal Bone examinations (*p* ≤ 0.02) (Table 4 and Fig. 2). The mean reductions in RT, with corresponding relative efficiency gains, were 2.9 min (20%) for CT Abdomen, 2.2 min (15%) for CT Total Body, 0.7 min (8%) for CT Head, and 2.5 min (22%) for CT Temporal Bone. Importantly, these categories also demonstrated notable reductions in median and 75th percentile RTs, indicating an improvement across the broader distribution of RTs. No significant changes were observed for CT Chest (−0.4 min, −3%; *p*-value = 0.07) and CT Sinus/Orbits (−0.1 min, −2%; *p*-value = 0.90).

**Table 4:** Impact analysis of radiologists’ RTs before and after implementation of an AI-aided DICOM standardization tool^a^

		CT Chest	CT abdomen	CT total body	CT head	CT sinus/orbits	CT temporal bone
Cases (N)	Before	4006	2426	1495	1702	1036	301
	After	2453	2590	2437	1449	1089	324
Cases per year (N)^b^		4306	3344	2621	2101	1417	417
Average RT^c^ (min)	Before	10.6	14.7	14.6	8.9	10.7	11.3
	After	11.0	11.8	12.4	8.1	10.8	8.8
Efficiency gain^d^ (absolute, min) [relative, %]		−0.4 [−3.3%]	2.9 [20.2%]	2.2 [15.1%]	0.7 [8.3%]	−0.1 [−1.6%]	2.5 [21.8%]
Efficiency gain^e^ annual (absolute, min)[h]		−1722.4 [−28.7]	9697.6 [161.6]	5766.2 [96.1]	1533.5 [25.6]	−141.7 [−2.4]	1045.8 [17.4]
Median RT (min)	Before	6.6	7.7	10.6	4.8	9.1	8.0
	After	6.6	6.7	9.9	4.5	8.3	7.5
Difference median RT (%)		0.3	−12.0	−6.8	−6.2	−8.8	−6.8
25th percentile (min)	Before	4.7	5.2	7.7	3.5	4.2	5.7
	After	5.1	4.8	7.5	3.2	5.4	5.4
75th percentile (min)	Before	9.2	26.2	13.6	14.0	12.9	9.8
	After	8.9	20.7	12.4	6.7	11.6	9.1
Mann–Whitney U	*p*-value	0.07	< 0.001	< 0.001	< 0.001	0.90	0.02

^a^ Calculations were performed using non-rounded values, the reported numbers in this table are rounded for clarity

^b^ Cases per year: based on the total number of cases before and after implementation over a cumulative time period of 18 months

^c^ RT: reading time

^d^ Efficiency gain: absolute and relative difference in average RT before and after AI tool implementation

^e^ Annual efficiency gain estimates: absolute efficiency gain^d^ multiplied by the annual case volume^b^ for each CT category

Extrapolation to annual case volumes estimated a total efficiency gain of 270 h per year across all CT categories, equivalent to approximately 34 eight-h working days. This estimate was derived from the annual efficiency gains calculated for each CT category, with the detailed calculations provided in Table 4. Because data were pooled across multiple NHS trusts, institutional-level gains may vary depending on local case volumes and distribution.

## Discussion

The aim of this study was to evaluate the labeling accuracy of an AI-aided tool for DICOM standardization and its impact on radiologists’ RTs. Labeling accuracy, evaluated across imaging modalities (CR and CT) and multiple anatomic regions, ranged from 83% to 100% for body part labels, 91% to 100% for plane labels, and 88% to 100% for protocol labels. A small proportion of studies/images remained unlabeled, likely due to data loss during anonymization rather than AI failure, and were excluded from calculations. The impact analysis revealed significant reductions in average RTs (*p*-value ≤ 0.02) for most study groups, including CT Abdomen, CT Total Body, CT Head, and CT Temporal Bone, corresponding to relative efficiency gains of 8% to 22%. Extrapolation to annual case volumes yielded an estimated total efficiency gain of 270 h per year across all CT categories, equivalent to approximately 34 eight-h working days. Because data were pooled across multiple NHS trusts, institutional-level gains may vary depending on local case volumes and distribution.

By improving the consistency and clarity of DICOM labels, this AI tool addresses long-standing challenges in imaging data management. Previous studies have identified that inconsistent or incomplete metadata hinders essential workflows, such as prior image retrieval, generation of hanging protocols, research data extraction, and cross-institutional data sharing. Such inconsistencies are also a major barrier to the effective deployment of AI applications in radiology [4]. Despite the rapid growth of AI applications in medical imaging, relatively little attention has been devoted to tools that enhance workflow processes. This is reflected in the notable absence of studies on this topic over the past decade. In particular, there is a critical gap in the current literature regarding the standardization and optimization of foundational infrastructure like DICOM metadata [4, 6, 11, 14, 15, 23]. Given this gap, comparing our results to prior research is challenging. However, the absence of previous studies further underscores the value of our work as the first peer-reviewed evaluation of this tool.

While these findings highlight the AI tool’s potential, the observed results should be interpreted with caution. Strengths of this evaluation include the use of a robust dataset of 648 cases containing 1925 images/series collected over nearly two years for the labeling accuracy analysis, encompassing two imaging modalities and a diverse range of body parts. In addition, the impact analysis was based on an extensive before-and-after analysis, including RTs of 10,966 cases before and 10,342 cases after AI tool implementation.

Several limitations warrant discussion. Regarding the labeling accuracy evaluation, the major limitation is the lack of independently established ground-truth labels against which the AI-generated DICOM labels could be compared. As a result, a sensitivity and specificity analysis could not be performed, and observer bias cannot be excluded. A multi-reader or consensus approach could further strengthen the robustness of the ground truth. Although the AI tool demonstrated very high DICOM labeling accuracy, minor inaccuracies were observed in body part labeling of CT studies, particularly in anatomically overlapping regions. This likely reflects the need for further finetuning of software threshold parameters to determine how partial body parts are classified. Additionally, while the tool reliably differentiated between non-contrast and contrast-enhanced studies, distinguishing specific enhancement phases proved more challenging, indicating a need for further algorithmic refinement.

Regarding the impact analysis, some additional limitations warrant consideration. First and foremost, the precise mechanism underlying the significant reductions in RTs could not be definitively established. The SDPs triggered by the AI tool were not directly assessed. Therefore, it remains an assumption that the observed efficiency gains were primarily driven by improved metadata standardization and associated improvements in hanging protocols.

Second, several methodological factors may have led to an underestimation of the tool’s true effect. Most importantly, the definition of “reading time” encompassed the entire period from opening the study to report closure, including both interpretation and reporting activities. This broader definition, combined with influences such as fatigue, workload variability, and workflow interruptions, introduces the risk of information bias. Although the large scale and diversity of the dataset reduce the likelihood that such factors fully explain the observed effects. In addition, a two-month transition period was arbitrarily chosen to allow radiologists to adapt to the standardized SDPs. A longer adaptation period may have reduced variability related to individual learning curves and SDP usage [27].

Third, the CT Chest group in the RT analysis requires particular attention. A substantial discrepancy in case numbers was observed before (*n* = 4006) and after (*n* = 2453; 39% fewer) AI implementation, despite using identical selection criteria. Several factors, such as temporal fluctuations in disease incidence, particularly in relation to the COVID-19 pandemic and its various waves, and contractual changes with participating clients or thoracic radiologists, may have contributed to this discrepancy. However, these factors alone are unlikely to fully account for the observed difference. While the exact cause of this imbalance remains unclear, we believe it is more likely to have negatively impacted the tool’s performance, rather than introducing bias in its favor. The lack of significant change in RTs in this group could also be attributed to the inherently simpler structure of the studies, the relative simplicity of prior imaging (e.g., CR chest studies), and/or the already accurate DICOM metadata available prior to AI-aided standardization.

Despite these considerations, this AI tool shows promising results. The potential benefits of this tool are not limited to radiologists. Standardized DICOM metadata may also facilitate workflows for non-radiology-trained users, including clinicians, emergency physicians, paramedics, students, and researchers [28]. In addition, the observed reduction in RT may translate into broader efficiency gains at the institutional level, particularly in high-volume settings [28].

Further research is needed to build upon the current findings. We hope that our study will serve as a foundation for future investigations in this area. Addressing the sources of bias identified in this study will be essential to ensure a more robust assessment of the impact of AI-aided DICOM standardization. Future studies should also evaluate broader clinical and institutional outcomes, including their application in more complex imaging modalities such as magnetic resonance imaging, the assessment of cost-effectiveness [28, 29], and the integration across different practice environments.

## Conclusion

This retrospective evaluation suggests that an AI tool can adequately standardize DICOM labels and may be associated with statistically significant reductions in radiologists’ RTs following implementation. By addressing long-standing challenges in imaging data management, such a tool has the potential to help optimize radiology workflows, benefiting both radiologists and other healthcare professionals.

## Supplementary information

ELECTRONIC SUPPLEMENTARY MATERIAL

## List of abbreviations

AI: Artificial intelligence
C-Spine: Cervical spine
CR: Computed radiography
CT: Computed tomography
DICOM: Digital imaging and communications in medicine
IRB: Institutional Review Board
NHS: National Health System (United Kingdom)
PACS: Picture archiving and communication system
RT: Reading time
SDP: Standard/smart display protocol
UK: United Kingdom
