# Supplementary material for: Evaluation of standardized DICOM labels assigned by a hybrid AI tool and its impact on radiologists’ reading times
Source: Eur Radiol. 2026 Jun 10;36(10):7535–45. doi: 10.1007/s00330-026-12670-w (PMC13574735; doi:10.1007/s00330-026-12670-w)
Supplement: Supplementary file 1 [file 330_2026_12670_MOESM1_ESM.pdf]

Evaluation of standardized DICOM labels assigned by a hybrid AI tool and its impact on radiologists' reading times

ELECTRONIC SUPPLEMENTARY MATERIAL

Software description

Publicly available patents were consulted due to the absence of peer-reviewed scientific articles. These patents were used exclusively to explain the operational principles of the ENDEX™ system, while maintaining the confidentiality of its proprietary model. We ensured that this description remained as objective and concise as possible. This tool is an AI-aided application positioned between image acquisition and PACS ingestion. It standardizes DICOM metadata by mapping inconsistent, incomplete, or non-standard entries to a consistent ontology.

According to US Patent 11,295,840 B2 ("Medical Scan Header Standardization System"), the system combines rule-based preprocessing of DICOM header fields with deep learning-based classification. This includes parsing existing metadata (e.g., modality, study description, series description, body part examined), analyzing pixel data using convolutional neural networks (CNNs) to identify for example anatomical regions, scan planes, and contrast phases when metadata is incomplete, and aligning results with controlled vocabularies for semantic consistency. [1]

US Patents 11,107,564 B2 and 11,669,791 B2 describe how the software can match imaging studies to corresponding reports using visual model inference and update accession numbers when mismatches are detected. This process supports linkage between image data and associated clinical records. [2; 3]

Supplementary Tables

Supplementary Table 1. Study group definition for labeling accuracy analysis based on DICOM body part and study description labels.

**Group name**	**Body part label**	**Study description label**[a]
CR Chest	Chest	XR chest
CR C-Spine[b]	C Spine, CSpine, C_Spine<br>Neck<br>Odontoid	XR cervical<br>XR mandible
CR Knee	Knee<br>Lower extremity	XR knee<br>XR knee left<br>XR knee right
CT Chest	Chest<br>Thorax	CT chest noncon[c]<br>CT chest<br>CT neck/chest
	Missing	CT chest/abdomen/pelvis<br>CT neck/chest/abdomen/pelvis
CT Abdomen	Abdomen<br>Abdomen/pelvis<br>Colon	CT abdomen/pelvis<br>CT abdomen/pelvis noncon
CT Total Body	CAP[d]<br>NCA[d]<br>NCAP[d]<br>Neck_CA[d]<br>Neck_CAP[d]	CT abdomen/pelvis<br>CT chest/abdomen/pelvis<br>CT neck/chest/abdomen<br>CT neck/chest/abdomen/pelvis
CT Head	Brain<br>Head	CT head<br>CT head noncon<br>CT head/neck<br>CT head/neck noncon
CT Face	Face<br>Facial bone, facial bones, F bones<br>Sinus, sinuses<br>Mandb, mandible<br>IAMS[e]<br>Missing	CT head noncon<br>CT head/neck noncon

[a] Radiology studies with either a body part label or study description label matching the assigned group.
[b] C Spine: cervical spine
[c] noncon: non-contrast enhanced
[d] (N)CA(P): (neck)-chest-abdomen(-pelvis)
[e] IAMS: internal auditory meatus

Supplementary Table 2. Study group definition for the impact analysis based on study description.

Group name	Study description[a]
CT Chest[b]	CT Thorax<br>HRCT[d] Thorax<br>CTPA[e]
CT Abdomen[b]	CT abdomen<br>CT abdomen/pelvis
CT Total Body[b]	CT Thorax. CT Abdomen<br>CT Thorax and CT abdomen<br>CT Thorax. CT colon.<br>CTPA. CT abdomen<br>HRCT Thorax. CT colon.
CT Head[c]	CT Brain<br>CT Head
CT Face	CT Facial bones<br>CT Facial bones/sinuses<br>CT paranasal sinuses complex<br>CT orbit
CT Temporal Bone	CT Temporal bone<br>CT IAM[f]<br>CT facial bones/sinuses. CT Temporal bone<br>CT facial bones/sinuses. CT IAM

[a] Raw data consisted of anonymized accession numbers, year and month of study, modality, study description and reading time duration in minutes.
[b] CT studies involving the thoracic and/or abdominal aorta were excluded to maintain homogeneity in the Chest, Abdomen and Total Body groups and to avoid overlap between them.
[c] CT head studies combined with CT intracranial vessels (e.g. stroke protocol) were excluded.
[d] HRCT: High resolution CT
[e] CTPA: CT pulmonary embolism
[f] IAM: internal auditory meatus

Supplementary Table 3. Detailed overview of deviations in AI-assigned DICOM labels identified in the labeling accuracy analysis.

Group name	Error type	Error frequency (number of images, N)	Label type	AI-assigned DICOM label	Visual evaluation of gold standard
CR Chest	n.a.	n.a.	n.a.	n.a.	n.a.
	n.a.	n.a.	n.a.	n.a.	n.a.
CR C-Spine[a]	Mislabeled	2	Plane	Mandible oblique	Lateral
	Mislabeled	1	Plane	Frontal	Lateral
CR Knee	Mislabeled	1	Plane	Intercondylar[b]	Lateral
	Mislabeled	2	Plane	Oblique	Lateral
	Mislabeled	2	Plane	Axial[c]	Lateral
	Mislabeled	2	Plane	Skyline[c]	Lateral
CT Chest	Mislabeled	7	Body part	Chest	Chest/abdomen
	Mislabeled	6	Body part	Neck	Chest
	Mislabeled	19	Protocol	CE Arterial	CE venous
	Mislabeled	4	Protocol	CE Venous	CE dual rule out
	Mislabeled	6	Protocol	CE Delay	CE late arterial/ early venous
	Missing	54	Body part	Empty	CAP
		1	Body part	Empty	Chest/abdomen
		1	Body part	Empty	Chest
CT Abdomen	Mislabeled	22	Body part	Abdomen	A cropped reconstruction of the basal lung fields was included as part of a CT abdomen study. The body part label was correctly assigned as "abdomen", and this label was assigned to series including the reconstruction in lung window of the basal lungs and small portion of the upper abdomen. Given that this reconstruction represents an atypical image, capturing only a limited section of

					both the chest and abdomen, and that the primary focus of the study was a CT abdomen, it was not considered an error and for sake of clarity excluded from the accuracy calculations presented in Table 2.
	Mislabeled	1	Protocol	Contrast enhanced venous phase	Oral contrast, no intravenous contrast enhanced.
	Missing	31	Body part	Empty	Abdomen/pelvis
CT Total Body	Mislabeled	2	Body part	Neck	Chest
	Mislabeled	17	Body part	Neck	NCAP
	Mislabeled	7	Body part	NCAP	NCA
	Mislabeled	3	Body part	NCAP	CAP
	Mislabeled	5	Body part	CAP	NCAP
	Missing	14	Body part	Empty	NCAP
CT Head	Mislabeled	20	Body part	Brain	Facial bones
	Mislabeled	7	Protocol	Non-contrast enhanced	Contrast enhanced
	Missing	7	Body part	Empty	Head
CT Face	Missing	22	Body part	Empty	Face

[a] C-Spine: cervical spine
[b] Intercondylar and frontal both categorized as "frontal"
[c] Skyline and axial both categorized as "axial"
[d] (N)CA(P): (neck)-chest-abdomen(-pelvis)
[e] CE: contrast enhanced

References

1 Lyman KU, A; Yao, L; Prosky, J; Poblenz, EC; Croswhite, C; Covington, B (2022) Medical Scan Header Standardization System. Enlitic Inc., United States

2 Poblenz EL, K; Croswhite, C (2021) Accession Number Correction System. Enlitic Inc., United States

3 Poblenz EL, K; Croswhite, C (2023) Accession Number Correction System and Methods for Use Therewith. Enlitic Inc., United States
